# Strategies for talent engagement and retention of Brazilian Nursing professionals

**DOI:** 10.1590/0034-7167-2022v75n6refl

**Published:** 2022-03-21

**Authors:** Francine Schlosser, Marcia Carvalho de Azevedo, Deborah McPhee, Jody Ralph, Hanna Salminen

**Affiliations:** I University of Windsor, Odette School of Business. Ontario, Canada.; II Universidade Federal de São Paulo. São Paulo, São Paulo, Brazil.; III Brock University, Goodman School of Business. Ontario, Canada.; IV University of Windsor, Faculty of Nursing. Ontario, Canada.; V Tampere University. Tampere, Finland.

**Keywords:** Nursing, Post-Pandemic, Talent Management, Engagement, Talent Retention

## Abstract

**Objective:**

To reflect on how human resource health managers and talent managers may engage and retain experienced nursing professionals in Brazil.

**Methods:**

Reflection based on studies on global and Brazilian-specific nursing professionals and retention, before and during the COVID-19 pandemic.

**Results:**

The pandemic worsened working conditions for all health professionals. Nursing professionals were particularly affected. Nurses have been viewed as “heroes” and “essential” frontline workers during the COVID-19 pandemic. However, despite the universal praise for their efforts, it seems uncertain if they were actually considered and managed like talent.

**Final considerations:**

In order to develop a sustainable healthcare system supported by sufficient experienced nursing talent, healthcare human resource managers and talent managers must develop and implement impactful nursing talent retention and engagement strategies. We highlight possible strategies targeting experienced nursing talent that will help to sustain the Brazilian healthcare system, post-pandemic.

## INTRODUCTION

COVID-19 has highlighted the importance of engaging and retaining experienced registered nurses who can be redeployed in emerging complex situations^(1)^. Pandemic demands have tested the professional engagement and commitment of experienced nursing professionals^(1)^. Increasingly vulnerable to stress and burnout, many experienced nurses intend to retire, leave the profession or move to roles that are less demanding^(2)^. While this sentiment has been expressed throughout the pandemic, the emergence of the Omicron variant has worsened this situation. Despite the global shortage of nurses in developed countries (eg., North America and Europe), until recently there has been no systematic approach to the retention and re-attraction of retiring nurses^(3)^. Indeed, developed countries are turning to less developed countries, such as Brazil, to source nursing talent. Consequently, Brazil’s ability to retain experienced nursing professionals is increasingly threatened by the global shortage of nurses.

Nursing in Brazil has a historical trajectory that is connected with a specific professional profile and hierarchies associated with health teams. Perspectives of gender, class and race/ethnicity have an impact on the nursing profession, a category that traditionally does not carry a high status in the health sector. The pandemic has increased historical inequalities and exposed the social invisibility of the category and its strenuous working conditions^(4)^. In a pandemic context, human resource health managers are dealing with additional challenges to attract and retain Brazilian nurse professionals, as the nursing profession has a significant workload, but has a lower status and wages, when compared with other health professionals.

In response to these talent challenges, we ask: ***How can human resource health managers and other talent managers engage and retain experienced nursing professionals in Brazil?*** As we consider the increasingly chaotic nature of careers in nursing, especially in a pandemic and uncertain environment, we build upon talent management theory to strategize effective talent retention and engagement of experienced nurses.

## METHOD

This is a reflection on Brazil’s challenges related to the management of experienced nursing professionals, who may leave Brazil to seek recognition as talent elsewhere in the world, or even may decide to change profession. We conducted a non-systematic search for studies that concerned the challenges facing the engagement and retention of experienced nurses. The online search took place between June 2021 and December 2021, using keywords such as nursing professionals, nursing shortage, global pandemic, nursing burnout. We began with a global search and then considered the experience of Brazilian nurses in a global context. The authors comprise a multi-disciplinary group of researchers, in the fields of nursing, management, and human resources management. Hence, the article builds on their varied clinical and work experience, and diverse academic fields of knowledge. The ensuing reflection provides suggestions for managing nursing talent grounded in both academic and practitioner literature.

## RESULTS AND DISCUSSION

### Brazilian Context

The Brazilian population is experiencing an accelerated aging process, a factor that has been steadily increasing the demand of various health services. This situation has significantly worsened during the COVID-19 pandemic. It is a worrisome context because clinical data and research results have shown that some COVID-19 patients have lingering effects that might require an ongoing need for a variety of health services, and in some cases for the rest of their lives^(4)^. In spite of the improvement of indicators of availability of health professionals (including nurses) and services, professionals usually experience a very heavy workload, coupled with insufficient resources^(5)^. An extremely demanding work environment during the pandemic particularly affected Brazilian nurses, faced with precarious working conditions^(6)^. In fact, Brazil represented a third of all deaths of nursing professionals by January 2021^(7)^.

### The Brazilian Healthcare System

Brazil has a complex and sophisticated health system that includes public and private health services. The public system is based on a Unified Health System (Sistema Único de Saúde, SUS), supported through taxes, that assures all Brazilians have a free and public health care service available. Parallel to the public health system there is also a private health system, that operates mainly with health insurances, and covered 48.1 millions of users in April 2021 (22 per cent of the population)^(8)^. The public system has four main types of services: Health Basic Units - Unidades Básicas de Saúde/UBS (deliver basic care at a neighborhood level), emergency care units (urgent care that delivers first care and if necessary forwards patients to hospitals), specialized medical assistance (patients forwarded by UBSs for specialized doctors) and hospitals. The number of public and private hospitals in 2020 was 6,642. This is a high number, but hospitals are mainly concentrated in big cities and with a significant variability of level and quality of services available. Nurses work in all these environments and are responsible, together with technical and assistant nurses, for the daily monitoring of patients^(9)^.

Brazilian nurses face a highly demanding work environment, with 41.5 per cent working more than 40 hours a week, with low wages (almost 50 per cent earned monthly salaries of less than USD 1,700 in 2013), and with more than half working in hospitals. Healthcare for the vulnerable and low-income population is mainly offered by public institutions, where working conditions are evaluated as “not adequate” by 41.6 per cent of nursing professionals^(5)^. Health assistance for the majority of the population is delivered by the public system, which faces considerable challenges to retain the best nursing professionals due to the competition with the higher salaries offered by the private health sector. The pandemic worsened working conditions for all health professionals and nursing staff was particularly affected by an increase in work demands, with extra shifts, a lot of patients to take care of, and many times, not having enough individual personal protection.

### Trajectory of Nursing Professionals

To meet the growing demand for health services, the number of nursing professionals has expanded considerably over the years, rising from 3.41 professionals per 1,000 inhabitants in 2001, to 11.65 in 2021, an increase of 341 per cent. This is all in spite of the fact that the population has only grown by 24.5 per cent in the same period. This phenomenon occurred as a result of a significant rise in the educational level of the Brazilian population, including an increase in the number of enrollments in both private (57.4 per cent) and public (35.6 per cent) higher education, which led to a rejuvenation process of the profession. It is important to mention that 80 per cent of the nurses attended some additional courses after graduating from university. In 2021 the number of nurses per 10,000 was 2.87, not far below WHO recommendations of 3.00 nurses to population ratio^(5)^.

In 2021, Brazil had 2,514,260 nursing professionals, comprising 614,526 nurses (24.6 per cent), 1,459,025 technical nurses (58.0 per cent), and 436,377 assistant nurses (17.4 per cent)^(7)^. To become a nurse a person must attend an undergraduate institution (university or faculty) and the course lasts four years. In Brazil, nursing is predominantly represented by younger workers (66.6 per cent are 40 years and below), and is female-dominated (86.2 per cent females). The majority of professionals (98.8 per cent) are Brazilians and nearly 50 per cent live in the Southeast region which is the most populated and developed part of the country. Almost 60 per cent are white, 31.3 per cent are mixed ethnicity and 0.3 per cent indigenous. Public institutions employ half (49.7 per cent) of the professionals^(5)^.

After graduation all nurses in Brazil are allowed to perform the same set of procedures and activities regardless of years of experience in the field. However, it is important to note that experienced nursing talent takes many years to develop. Benner^(10)^ noted the following five stages of evolution in skills development of nurses:

1. inexperienced novice (student nurse);
2. advanced beginner (recent graduate) with a need for an experienced mentor to help set priorities;
3. competent stage (2-3 years) where the nurse is able to prioritize tasks by using past experiences;
4. proficient stage at which the nurse is able to see the situation in its entirety with a holistic understanding;
5. expert, where the nurse has an extensive knowledge of complex patient situations.

Although these levels of expertise are somewhat universal, an experienced nurse may “drop” a level if the setting changes or the patient population changes (e.g., a surgical nurse moving to the intensive care unit or a community nurse moving to hospital). The redeployment of nursing professionals to the uncertain, dynamic and demanding COVID-19 patient wards required that nurses surmount a significant learning curve. Such difficult working conditions have challenged human resource healthcare managers to identify and manage nursing talent, thereby supporting sustainable nursing careers.

### The Challenges of Developing Nursing Talent

Nurses have been viewed as “heroes” and “essential” frontline workers during the COVID-19 pandemic. However, despite the universal praise for their efforts, it seems uncertain if they were actually considered and treated like talent^(1)^. Vaiman et al.^(11)^ defined talent management as “a set of organizational processes designed to attract, develop, mobilize, and retain individuals with high levels of human capital and ensure their deployment in roles which are pivotal to organization success”.

Meyers et al.^(12)^ recommended that organizations employ an exclusive approach during a war for talent to identify, attract, and retain talented individuals. Exclusive talent rests on the notion that talent is rare and exists only in a small number of elites with strategic impact^(13)^. Experienced nurses represent an important source of highly trained and experienced talent, but nurses were not managed like exclusive talents during the pandemic, where many experienced nurses were expected to do their jobs without enough personal protective equipment (PPE), lagged vaccinations, and no structured organizational support. If they were not treated as exclusive talents, then one might think that health talent managers would at least treat all nurses with an inclusive approach, which involves recognizing and developing the character strength and particular talent(s) of all nurses with appropriate training and development. However, redeployed nurses were offered little training for a pandemic situation that demanded new protocols, technologies and practices. Talent management practices have focused universally on the recruitment and not on the retention of nurses. This means that experienced nurses are replaced by a pipeline of inexperienced young nurses often recently graduated. In Brazil and other developing countries, this is particularly true, given the youthful average age of nursing professionals is 35.6^(14)^ compared to the average age in developed countries like Canada which reports an average age of 46^(15)^.

## FINAL CONSIDERATIONS

In order to develop a sustainable healthcare system supported by sufficient experienced nursing talent, healthcare human resource managers (HRM) and talent managers (TM) must develop and implement impactful nursing talent retention and engagement strategies. In the following sections we highlight possible strategies targeting experienced nursing talent.

### Talent Retention Strategies

HRM can recognize nursing professionals as talent and help nurses feel appreciated by engaging experienced nurses close to retirement and re-engaging retirees. A recent study concluded that experienced nurses with longer tenure with the organization were more affectively committed and had stronger intentions to remain^(16)^. This underscores the need to continue to engage healthcare workers over the long-term.

Healthcare talent managers can also recognize nursing talent by providing coaching to deal with chaotic uncertainty in their careers and environment and by further developing their professional competencies, even though these nurses are older employees. Employees will resist change - because it disrupts the fractal pattern of their lives, but ongoing learning will promote their adaptive capacities^(17)^. To this end, healthcare talent managers should encourage a positive approach to uncertainty by developing “decision and counseling framework that helps clients deal with change and ambiguity, accept uncertainty and inconsistency and utilize the non-rational and intuitive side of thinking and choosing”^(18)^. For example, there is evidence that older nursing professionals with high levels of resilience are more likely to continue working until retirement age or even beyond compared to those older nurses with lower levels of resilience^(19)^.

As nurses try to deal with stress and burnout, they will consider alternatives that include retiring, seeking a less stressful job in the same workplace, seeking accommodation from the healthcare employer, and considering offers from other institutions. To combat these alternatives, healthcare talent managers should consider providing experienced nurses with occupational development and increased flexibility. Training is critical to ensuring career opportunities are available to foster retention of experienced nursing talent. In a recent study^(20)^, noted the importance of work-family balance, facilitated through individual consideration for work schedules to be key to retention.

Compensation is another issue that has contributed to stress and burnout. Nurses have recently in Brazil have historically low wages, especially when compared with developed countries, yet they have been complaining throughout the pandemic the need to be paid more because of the dangers they are faced, the longer working hours, isolated from their families to ensure their safety, and the emotional strain of dealing with continuous death and the roar of anti-vaxxers downplaying the urgency of this issue. The unfairness in the system only creates further angst, and a lack of feeling underappreciated, which manifests itself in feelings of stress and burnout^(21)^. In line with equity theory, it is imperative that more fair ways of rewarding those on the front line be considered immediately to avoid the further degradation of the profession and the ability to retain the talent already in place. As well, paying large hiring bonuses only keeps the problem moving around because when nurses leave one hospital for another, this pits the public healthcare institutions against private healthcare institutions and threatens patient care and overall Brazilian healthcare sustainability. Nothing is actually accomplished except to leave those who have put in the time feeling neglected and forgotten.

Understanding the perspective of experienced nursing talent requires that healthcare talent managers understand their vocational calling (e.g.,^(22-23)^, their need to be appreciated as talent, and the stress and burnout they must overcome in the face of chaotic uncertainty. Healthcare talent managers can develop nurses’ professional calling by supporting the development of nurses’ career resilience and providing them with adequate resources to do their jobs. These might include operational changes such as ensuring adequate staffing levels and supportive policy changes such as providing proper personal protective equipment and including nurses in the scheduling decisions for redeployment. This will help to keep the healthcare mission alive for key nursing talent.

### Talent Engagement Strategies

Talent engagement strategies are needed in order to avoid the alienation involved as feelings of appreciation are threatened by stress and burnout. When strenuous working conditions affect calling, talent engagement strategies are required. The development of these aspects can be promoted through access to coaches, counseling, and institutional support systems. Valencia et al.^(24)^ found that experienced nurses aged 31 to 49 years old valued working for autonomy and respect, camaraderie with peers, and providing for their families. Healthcare talent managers might consider how to relieve nurses deployed in particularly stressful nursing contexts by occasional redeployment to less stressful situations, and by providing them with regularly scheduled paid personal mental health days away from the workplace. Healthcare talent managers must also give voice to experienced nurses by engaging them in policy-making, perhaps through focus groups or specialized events that build camaraderie and innovation^(25)^.

With advances in technology related to patient care, older experienced nursing professionals may be reluctant to continue to engage in today’s workplace^(24)^. To stem this potential loss of experienced talent, it is important to build confidence in this talent through thoughtful training. Older workers learn better by showing how to do things, as opposed to methods of online learning desired by younger workers. Care must be taken to cater to each age group appropriately to achieve success and policies must be accompanied by supportive supervision^(26)^.

Inclusive talent management strategies must also provide targeted development and support for nurses who are from indigenous minorities in Brazil because, due to their previous experience and familiarity with indigenous culture and tradtions, they may be better prepared to deliver nursing services to remote indigenous populations. Currently, only .3 per cent of nurses are indigenous^(5)^, and it would seem important to not only cultivate their individual talents for retention, but also to utilize their experiences to understand how to better recruit this demographic for a sustainable healthcare system that serves all Brazilians.

Building upon this literature review, this reflection paper has presented an overview of the changing perspectives of nursing professionals as they face waves of uncertainty, most recently tested through the COVID-19 pandemic. We described talent management strategies for retaining and engaging the performance of experienced nurses, post-pandemic. We discussed the importance of equity theory and fairness in the distribution of rewards.

However, this reflection requires further study and empirical testing. We recommend a mixed method approach to better understand these complex social phenomena^(27)^ including collecting qualitative and quantitative data from primary sources and sourcing large secondary databases with existing data that present opportunities for machine learning (AI) to predict turnover. Future researchers should also ensure that they seek input from policymakers and healthcare leaders in Brazil who can weigh-in on the research and help disseminate findings of scientific significance to the public. When it comes to talent management practices, future research should investigate the perspectives of both nursing professionals and healthcare organizations. For example, it has been argued that there is a need to examine both the actual talent management practices, as well as how employees perceive those practices^(28-29)^. Furthermore, future studies should also investigate possible discrepancies between the healthcare organizations’ talent management discourse and actual practices^(28)^.
